# Role of *Streptococcus pneumoniae OM001* operon in capsular polysaccharide production, virulence and survival in human saliva

**DOI:** 10.1371/journal.pone.0190402

**Published:** 2018-01-02

**Authors:** Zuleeza Ahmad, Richard M. Harvey, James C. Paton, Alistair J. Standish, Renato Morona

**Affiliations:** Research Centre for Infectious Diseases, Department of Molecular & Cellular Biology, School of Biological Sciences, The University of Adelaide, South Australia, Australia; Instituto Butantan, BRAZIL

## Abstract

*Streptococcus pneumoniae* is the leading cause of community-acquired pneumonia in all ages worldwide, and with ever-increasing antibiotic resistance, the understanding of its pathogenesis and spread is as important as ever. Recently, we reported the presence of a Low Molecular Weight Tyrosine Phosphatase (LMWPTP) Spd1837 in the pneumococcus. This protein is encoded in an operon, *OM001* with two other genes, with previous work implicating this operon as important for pneumococcal virulence. Thus, we set out to investigate the role of the individual genes in the operon during pneumococcal pathogenesis. As LMWPTPs play a major role in capsular polysaccharide (CPS) biosynthesis in many bacteria, we tested the effect of mutating *spd1837* and its adjacent genes, *spd1836* and *spd1838* on CPS levels. Our results suggest that individual deletion of the genes, including the LMWPTP, did not modulate CPS levels, in multiple conditions, and in different strain backgrounds. Following *in vivo* studies, Spd1836 was identified as a novel virulence factor during pneumococcal invasive disease, in both the lungs and blood, with this protein alone responsible for the effects of operon’s role in virulence. We also showed that a deletion in *spd1836*, *spd1838* or the overall *OM001* operon reduced survival in human saliva during the conditions that mimic transmission compared to the wildtype strain. With studies suggesting that survival in human saliva may be important for transmission, this study identifies Spd1836 and Spd1838 as transmission factors, potentially facilitating the spread of the pneumococcus from person to person. Overall, this study hopes to further our understanding of the bacterial transmission that precedes disease and outbreaks.

## Introduction

*Streptococcus pneumoniae* (the pneumococcus) predominantly colonizes the nasopharynx as a commensal in healthy individuals [1]. However, the bacteria can transition to be an opportunistic pathogen, leading to diseases with significant morbidity and mortality such as pneumonia, bacteremia and meningitis. By blocking the colonization or carrier state with widespread immunization, rates of transmission within the community for the strains that are included in the vaccine formulations have declined and this in turns provides herd immunity for the unvaccinated populations [2, 3]. These epidemiological studies have also shown that older populations mainly acquire the pneumococcus from colonized children [4]. Therefore, this suggests that the vaccine exerts its efficacy by limiting the spread between immunized individuals and it is possible to target a specific step in pneumococcal pathogenesis which is the colonization stage.

Much of our work has previously focused on determining the role that tyrosine phosphorylation plays in the virulence of *S*. *pneumoniae* [5–8]. We have recently characterized Spd1837 as a protein tyrosine phosphatase (PTP) of the Low Molecular Weight Protein Tyrosine Phosphatase family (LMWPTP) that may interact with proteins associated with pneumococcal metabolism (Ahmad *et*. *al*., submitted for publication). LMWPTPs are also widely established to play a role in regulating capsular polysaccharide (CPS) and exopolysaccharide (EPS) biosynthesis [9].

In the chromosome of the serotype 2 *S*. *pneumoniae* strain D39, *spd1837* is co-transcribed together in the *OM001* operon with the upstream translocase subunit YajC (Spd1838), and a downstream hypothetical protein (Spd1836) (S1 Fig). While the operon is conserved across approximately 90% of pneumococcal strains with available genome sequence, little is known concerning the function of Spd1836 and Spd1838. Spd1836 consists of one conserved motif, the Membrane Occupation and Recognition Nexus (MORN) repeats. Despite being found in all domains of life and some viruses, very little is known about the MORN motif function. Based on limited studies conducted in apicomplexan parasites and *Arabidopsis*, the MORN motifs confer the ability of lipid binding, however, the subsequent role differs between species from regulating cell size to cell budding [10]. On the other hand, Spd1838 homologs have only been studied in Gram negative bacteria. In *E*. *coli*, YajC participates in Sec-dependent secretion by forming a complex with SecDF and YidC which may associate with the SecYEG and SecA ATPase to improve protein translocation efficiency [11]. Although the Sec-dependent pathway has been extensively studied, the precise role of SecDF-YidC-YajC complex is largely unknown.

Using a differential fluorescence induction (DFI) technique, the *OM001* operon was previously identified to be significantly upregulated in several *in vitro* conditions that mimic infection. Subsequent deletion of this operon severely attenuated the ability of the pneumococcus to cause infection in multiple *in vivo* infection models [12], however, the role of the individual genes of the operon remained unknown.

In a recent study, Verhagen *et*. *al*. [13] conducted a genome-wide negative selection screening using Tn-seq and found 147 genes potentially required for the pneumococcal survival and growth in human saliva. Of these, two out of the three genes from the *OM001* operon (*spd1836* and *spd1837*) were identified. Indirect evidence from studies in humans suggests saliva is a possible medium for person-to-person spread [14]. Not only could live pneumococci be isolated and cultured from human saliva [15], saliva culture was also found to be a more robust and sensitive method for detecting the bacteria compared to conventional and the more invasive methods of trans-nasal and trans-oral swabs [16]. Verhagen *et*. *al* [13] have shown that the pneumococcus could survive and even grow in pure human saliva in 24 hours period. This highlights the extreme ability of the pneumococcus to adapt to different environments, namely the nasopharynx and potentially the oropharynx during the colonization step of pneumococcal pathogenesis.

Although transmission is the important first step that precedes carriage and disease (in fact none of pneumococcal disease states facilitate contagion [17]), pneumococcal factors that foster transmission are not well characterized due to a lack of tractable models to study this process until recently [18]. Indeed, pneumococcal disease occurrence is directly linked to the strains circulating in carriage [19]. Transmission is thought to require close contact, such as between individuals within the same households or day care centre [20, 21]. While it is generally accepted that the pneumococcus is a human-obligate pathogen with no known environmental or animal reservoir, evidence accumulating is that the bacteria can survive outside of human host. For instance, rehydrated pneumococci were able to infect mice after being left desiccated for four weeks [22].

This study set out to investigate the role of the *OM001* operon in CPS biosynthesis, virulence and survival in human saliva. While there was a minimal role for the operon in CPS production, we have shown that the operon is important for the ability of *S*. *pneumoniae* to cause invasive disease and the ability to survive in human saliva. Specifically, we have identified Spd1836 as a previously uncharacterized virulence factor, while Spd1836 and Spd1838 are essential for the pneumococcal survival in human saliva at 25°C, a condition to mimic how the bacteria would survive outside of the human body during transmission. With ever-increasing antibiotic resistance, the continued identification of factors important for the virulence and transmission of the pneumococcus is critical to identify new targets for the development of antimicrobials.

## Materials and methods

### Growth media and growth conditions

*S*. *pneumoniae* strains (listed in Table 1) were routinely grown either in Todd-Hewitt broth with 1% Bacto yeast extract (THY) at 37°C as indicated or on Columbia blood agar (BA) plates supplemented with 5% (v/v) horse blood and grown at 37°C in 5% CO_2_ or, for mouse challenge, in serum broth (10% heat-inactivated horse serum in nutrient broth). Where appropriate, antibiotics were supplemented at the following concentrations: streptomycin at 150 μg mL^-1^, kanamycin at 200 μg mL^-1^ and gentamycin at 10 μg mL^-1^.

**Table 1:** List of strains used.

Strain	Description/antibiotic resistance^a^	Source/reference
D39	Sm	[8]
D39*spd1837*::*janus*	Km	(Ahmad *et*. *al*., submitted for publication)
D39Δ*spd1837*	Sm	(Ahmad *et*. *al*., submitted for publication)
D39Spd1837_C8S_	Sm	This work
D39Δ*spd1836*	Sm	This work
D39Δ*spd1838*	Sm	This work
D39Δ*OM001*	Sm	This work
D39Δ*OM001*::*janus*	Km	This work
D39Δ*OM001*::*OM001*^*+*^	Sm	This work
WU2	Sm	This work
WU2*spd1837*::*janus*	Km	This work
WU2Δ*spd1837*	Sm	This work

^a^ Sm, Streptomycin; Km, Kanamycin

### Construction of chromosomal mutation in *S*. *pneumoniae* D39

Markerless, non-polar mutant strains were constructed in a serotype 2 D39 streptomycin resistant strain and serotype 3 WU2 streptomycin resistant strain essentially as previously described [8]. First, the Janus cassette was used to target and replace the *spd1837* operon region in D39 and WU2 background strains [23]. Then the D39*spd1837*::*janus* strain was transformed with PCR products containing the in-frame deletion or point mutation in *spd1837*, or deletion in *spd1836*, *spd1838* or *OM001*. Additionally, PCR products containing the in-frame deletion of *spd1837* was also transformed into WU2*spd1837*::*janus* strain. All oligonucleotides used are listed in S1 Table. Transformations were carried out as described previously [24]. To create the *OM001* complemented strain, firstly, the 2 kb region upstream of the deleted *OM001* operon was amplified using the primers ZA3 and ZA16 and the 2 kb region downstream of the deleted *OM001* operon was amplified using the primers ZA4 and ZA19. These two PCR products and the amplified Janus cassette were combined and amplified again using just the primers ZA3 and ZA4. The approximately 2.4 kb PCR product was then used to transform D39Δ*OM001*. The transformants were selected on kanamycin plates, resulted in the intermediate strain, D39Δ*OM001*::*janus*. Next, the *OM001* operon region including 1 kb of flanking genomic DNA from D39 was amplified using the primers ZA36 and ZA37. This product was then used to transform D39Δ*OM001*::*janus* to replace the Janus cassette with the wild type copy of the *OM001* operon. The successful transformants were selected on streptomycin plate and one of them was sequenced and verified to have acquired the *OM001* operon back and this strain is called D39Δ*OM001*::*OM001*^*+*^.

### The production of polyclonal antibodies against Spd1837

Antibodies were raised against Spd1837 (purified protein > 95% pure as determined by Coomassie-stained SDS-PAGE (Ahmad *et*. *al*., submitted for publication)) (Institute of Medical and Veterinary Science, Veterinary Services (Gilles Plain, SA, Australia)) in rabbits. The antiserum was produced under the National Health and Medical Research Council (NHMRC) Australian Code of Practice for the Care and Use of Animals for Scientific Purposes and was approved by the University of Adelaide Animal Ethics Committee. The crude antibodies were enriched and affinity-purified using purified Spd1837 before being stored at -20°C in 50% (v/v) glycerol [25].

### SDS-PAGE and Western immunoblotting

The whole cell bacterial lysates were prepared from cultures grown in THY to an OD_600nm_ of approximately 0.3 and then subjected to SDS-PAGE and Western immunoblotting as described previously [26]. The concentrations of primary antibodies used were as follows; mouse anti-phosphotyrosine 4G10 antibodies (Bio X Cell) and mouse anti-CbpA at 1/5000 dilution, and rabbit anti-CpsD, rabbit anti-CpsB and rabbit anti-Spd1837 at 1/500 [27].

### Uronic acid assay

CPS was prepared from the indicated strains grown either aerobically (BA at 37°C with 5% CO_2_) or anaerobically (BA at 37°C with 5% CO_2_ in a BD GasPak™ Anaerobic Jar (Becton, Dickinson and Company)). The uronic acid assay was performed as described previously [7, 26]. Levels were related back to a standard curve of D-glucuronic acid (Sigma Aldrich). Differences in CPS levels were analyzed by one-way analysis of variance (ANOVA) with Dunnett’s post-hoc test.

### Mouse infection model

This study was carried out in strict accordance with the recommendations in the Australian Code of Practice for the Care and Use of Animals for Scientific Purposes (7th Edition (2004) and 8th Edition (2013)) and the South Australian Animal Welfare Act 1985. The protocol was approved by the Animal Ethics Committee at The University of Adelaide (approval number S/2013/053). Outbred 5-to-6-week-old female CD1 (Swiss) mice were used in all animal experiments. For intranasal (i.n.) challenge, mice were anesthetized by intraperitoneal (i.p.) injection of pentobarbital sodium (Nembutal; Rhone-Merieux) at a dose of 66 μg per g of body weight, followed by i.n. challenge with 50 μL of bacterial suspension containing approximately 1×10^7^ CFU mL^-1^ bacteria in serum broth. The challenge dose was confirmed retrospectively by serial dilution and plating on BA. Mice were euthanized by CO_2_ asphyxiation at the 48 hr post-challenge. Blood was collected by syringe from the posterior vena cava. The pleural cavity was lavaged with 1 mL sterile PBS containing 2 mM EDTA introduced through the diaphragm. Pulmonary vasculature was perfused by infusion of sterile PBS through the heart. Lungs were subsequently excised into 2-mL vials containing 1 mL sterile PBS and 2.8-mm-diameter ceramic beads for CFU counts. To obtain unattached pneumococci, the nasopharynx was subjected to lavage by insertion of a 26-gauge needle sheathed in tubing into the tracheal end of the upper respiratory tract and injection of 1 mL 0.5% trypsin–1×PBS through the nasopharynx. Additionally, the upper palate and nasopharynx were excised and placed into 2-mL vials containing 1 mL sterile PBS and 2.8-mm-diameter ceramic beads to obtain attached pneumococci. CFU counts for both the nasal wash and nasal tissue samples were combined to determine the total number of bacteria in the nasopharynx. Lung and nasopharyngeal tissues were homogenized using a Precellys 24 tissue homogenizer (Bertin Technologies) at 3 cycles of 30 s and 5,000 rpm. 40 μL aliquots of lung homogenate, nasopharyngeal tissues homogenate and pleural lavage, and 20 μL aliquots of blood were serially diluted and plated on BA supplemented with gentamycin to determine the number of CFU in these niches. Data were analyzed using non-parametric Mann-Whitney test. The incidence of pneumococcal invasion into the lungs and blood of mice were compared using two-tailed Fisher’s exact test.

### Evaluation of the survival of *S*. *pneumoniae* strains in human saliva

The University of Adelaide Human Research Ethics Committee approved the study protocol and the written informed consent form with approval number of H-2016-224. Saliva collection and *S*. *pneumoniae* survival tests were conducted essentially as described by Verhagen *et*. *al*. [13] with a few modifications. The additional criteria for recruiting participants include ‘currently a non-smoker’ and ‘no respiratory or periodontal disease or infection’ as smokers and individuals with such disease or infection were shown previously to have human leukocyte elastase in their saliva and therefore is not representative of general, healthy population [28, 29]. Briefly, fasting saliva of the donors was pooled and centrifuged at 16,000 *g* at 4°C for 15 minutes. The supernatant was sterilized by ultrafiltration with 0.45 μm Minisart filters (Sartorius Stedim Biotech). Before inoculation in saliva, the strains were grown in THY for 2 hr, diluted to a starting concentration of 1 × 10^6^ CFU mL^-1^ and washed twice in sterile PBS. The bacteria was incubated with at least 500 μL saliva at two conditions: 37°C with 5% CO_2_ (representing in-host carriage) and 25°C without CO_2_ (representing transmission). At t = 0, t = 3, t = 22, and t = 24 hr, samples were taken for CFU count. The number of bacteria at specific time point was enumerated by plating serial dilutions on BA plates. Experiments were performed in duplicates and repeated three times independently. Statistical differences between survival of *S*. *pneumoniae* in multiple dilutions of saliva were assessed by a one-way ANOVA and Dunnett’s post hoc tests.

## Results

### The proteins encoded in *OM001* operon do not play a role in CPS regulation

In another study, we showed that the Spd1837 was a PTP from the LMWPTP family (Ahmad *et*. *al*., submitted for publication). As a number of LMWPTPs modulate CPS and EPS biosynthesis, we investigated if Spd1837 and the co-transcribed genes encoding Spd1836 and Spd1838 played a role in the regulation of CPS in *S*. *pneumoniae*. Separate non-polar markerless deletion mutations in *spd1836*, *spd1837*, *spd1838* and of all three genes of the *OM001* operon were constructed in the chromosome of D39. We also constructed an in-frame unmarked point mutant (D39Spd1837_C8S_) which would not have any phosphatase activity. The strains (D39Δ*spd1837*, D39Spd1837_C8S_, D39Δ*spd1838*, D39Δ*spd1836* and D39Δ*OM001*) showed similar growth profiles to the parental strain D39 (S2 Fig).

**Fig 1:**
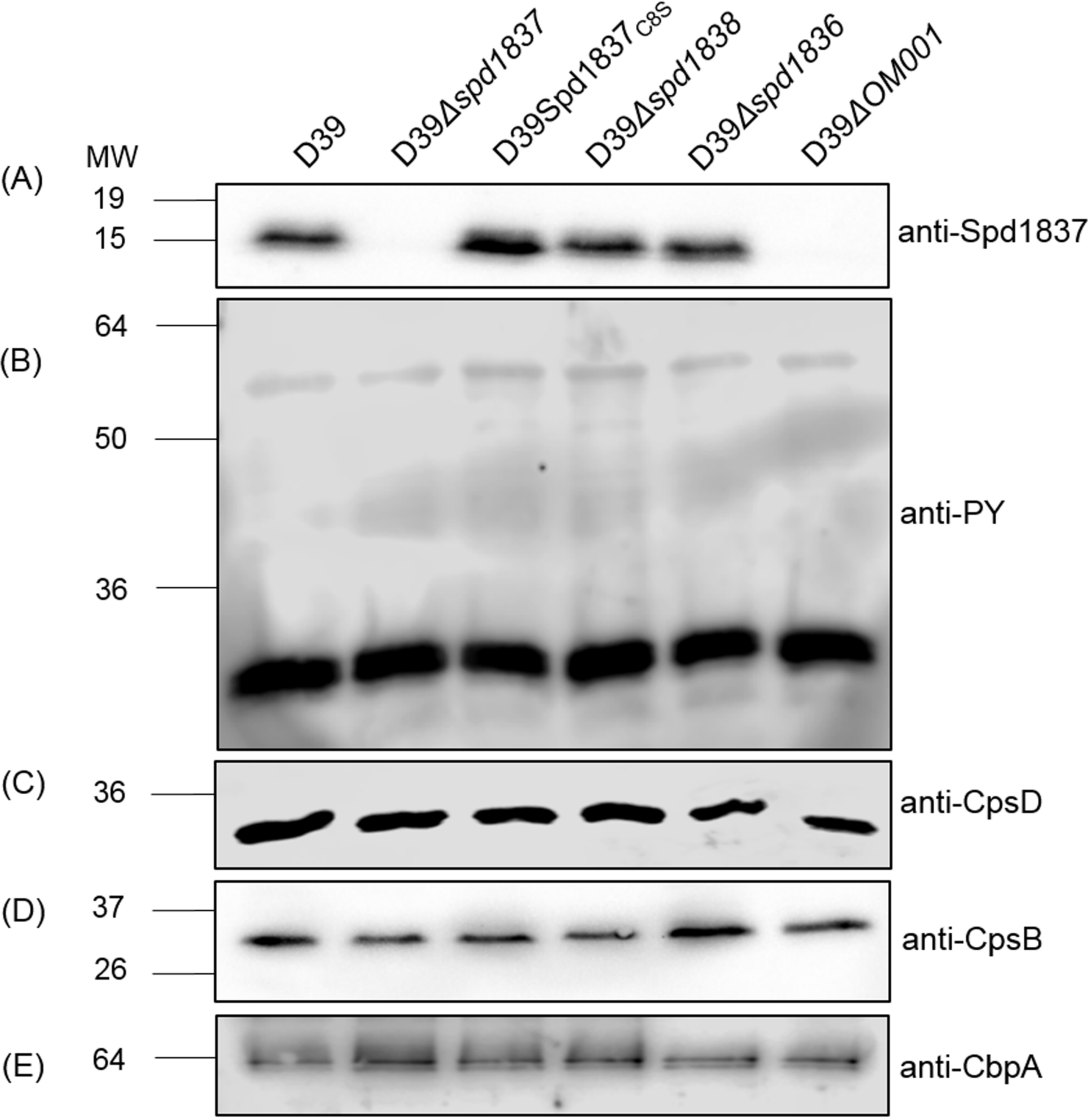
Proteins encoded by *OM001* operon do not alter tyrosine phosphorylation of CpsD. Proteins from whole-cell lysates from D39, D39Δ*spd1837*, D39Spd1837_C8S,_ D39Δ*spd1838*, D39Δ*spd1836* and D39Δ*OM001* cells were separated by SDS-PAGE, and Western immunoblotting was undertaken with anti-Spd1837 (A), anti-CpsD (B), anti-phosphotyrosine (PY) (C), anti-CpsB (D) and anti-CbpA (E). MW, molecular weight (in kDa). The arrow on (C) indicates a band corresponds to CpsD.

Western immunoblot analysis with an antibody against Spd1837, showed that D39Δ*spd1837* and D39Δ*OM001* did not produce Spd1837 while D39Spd1837_C8S_ still had the mutant form of Spd1837 produced at a level equivalent to the wildtype, as did D39Δ*spd1838* and D39Δ*spd1836* (Fig 1A). As tyrosine phosphorylation of CpsD is important for the CPS regulation in the pneumococcus [30, 31], we analyzed the overall tyrosine phosphorylation profiles of the mutant strains. All six strains had similar levels of overall tyrosine phosphorylation, specifically of CpsD (Fig 1B and 1C), indicating that at least under the growth condition used, Spd1836, Spd1837 and Spd1838 had no detectable effect on protein tyrosine phosphorylation. Additionally, the expression of the other known PTP in the pneumococcus, CpsB was also similar in these strains (Fig 1D). As a loading control, the expression of the choline-binding protein A (CbpA) was also checked and this verified that similar amount of proteins were loaded into each lane (Fig 1E).

**Fig 2:**
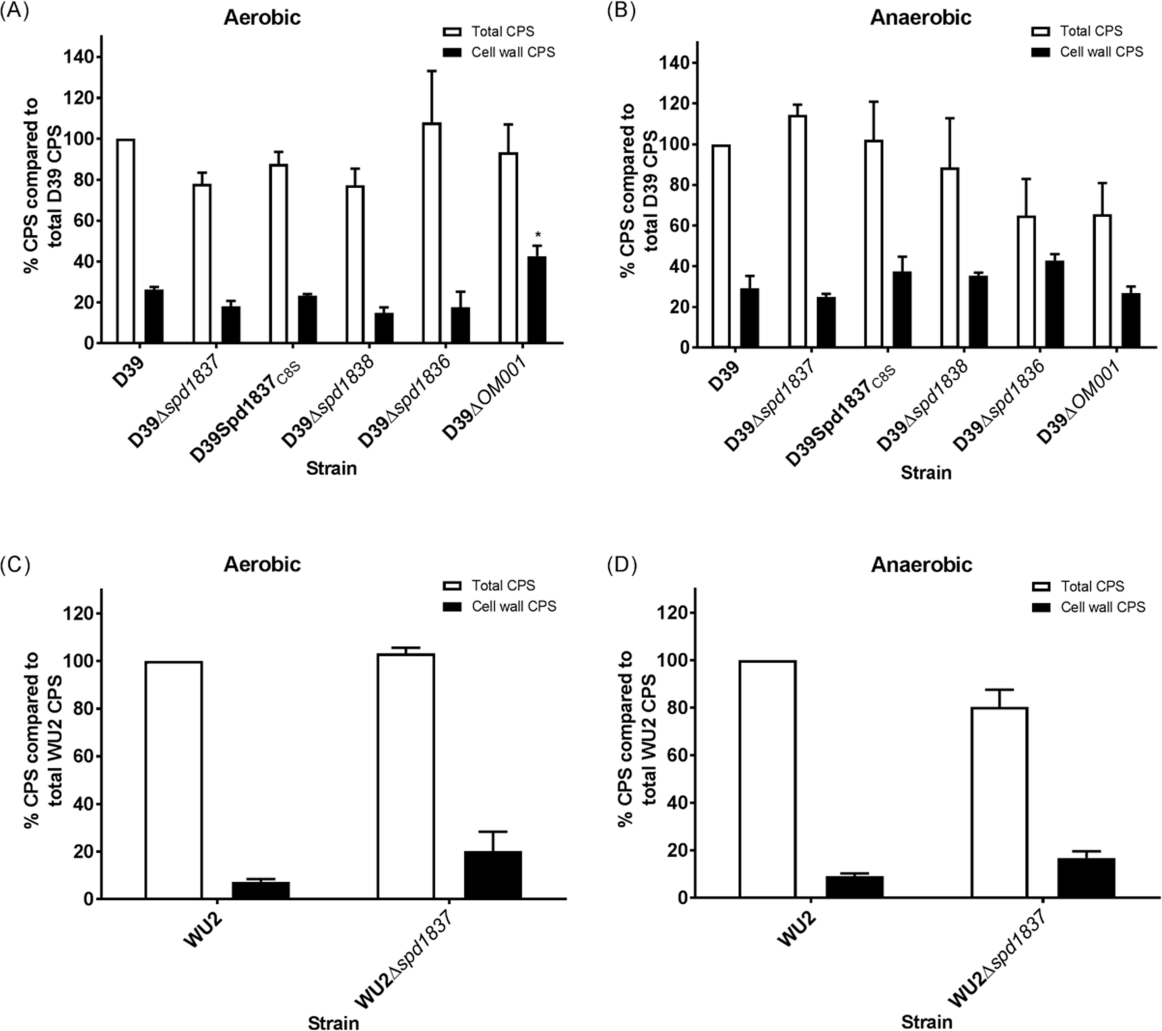
CPS production by D39 and WU2 strains. CPS was prepared from equal numbers of bacterial cells of D39, D39Δ*spd1837*, D39Spd1837_C8S_, D39Δ*spd1838*, D39Δ*spd1836*, and D39Δ*OM001* grown either aerobically (A) or anaerobically (B) and WU2 and WU2Δ*spd1837* grown either aerobically (C) or grown anaerobically (D). The CPS level was determined by uronic acid assay as described in Materials and Methods. The white bars represent the total CPS produced by various mutants as a percentage of total D39 CPS ((A) and (B)) or total WU2 CPS ((C) and (D)). The black bars represent the cell wall-associated CPS produced by mutants as a percentage of total D39 CPS ((A) and (B)) or total WU2 CPS ((B) and (C)). Bars represent means from three independent replicates while the error bars represent the standard error.

We then investigated whether these mutations modulated the synthesis of the CPS, using the uronic acid assay as described in the Materials and Methods. There was no significant difference in the amount of both total and cell wall-associated CPS produced by D39, D39Δ*spd1837*, D39Spd1837_C8S,_ D39Δ*spd1838* and D39Δ*spd1836* while the operon deletion mutant, D39Δ*OM001* had a slightly higher cell wall-associated CPS compared to that of the wildtype D39 strain under aerobic condition (Fig 2A). When we investigated CPS biosynthesis in anaerobic conditions, the overall total and cell wall-associated CPS levels of all strains were increased by approximately 20%, as previously observed [32]. However, there was no significant effect of mutating *spd1836*, *spd1837* or *spd1838* individually or together either on total or cell wall-associated CPS synthesis (Fig 2B).

Serotype 3 strains produce CPS via synthase-dependent mechanism [33], compared to the Wzy-dependent mechanism in serotype 2 and all other strains except serotype 37 [34–36]. This implies that Spd1837 is the only identified PTP in this serotype as it does not possess CpsB [33]. Thus, in order to investigate if Spd1837 played a role in CPS biosynthesis in this background, we also constructed a *spd1837* deletion in the serotype 3 strain WU2. Similar to in D39, there was no significant difference in CPS levels between WU2 and WU2Δ*spd1837*, either when the bacteria were grown aerobically (Fig 2C) or anaerobically (Fig 2D). This suggests that Spd1837 plays no role in the regulation of CPS biosynthesis in two serotypes of *S*. *pneumoniae* that synthesize CPS via two different mechanisms.

### Contribution of Spd1836, Spd1837 and Spd1838 to virulence in mouse model of infection

**Fig 3:**
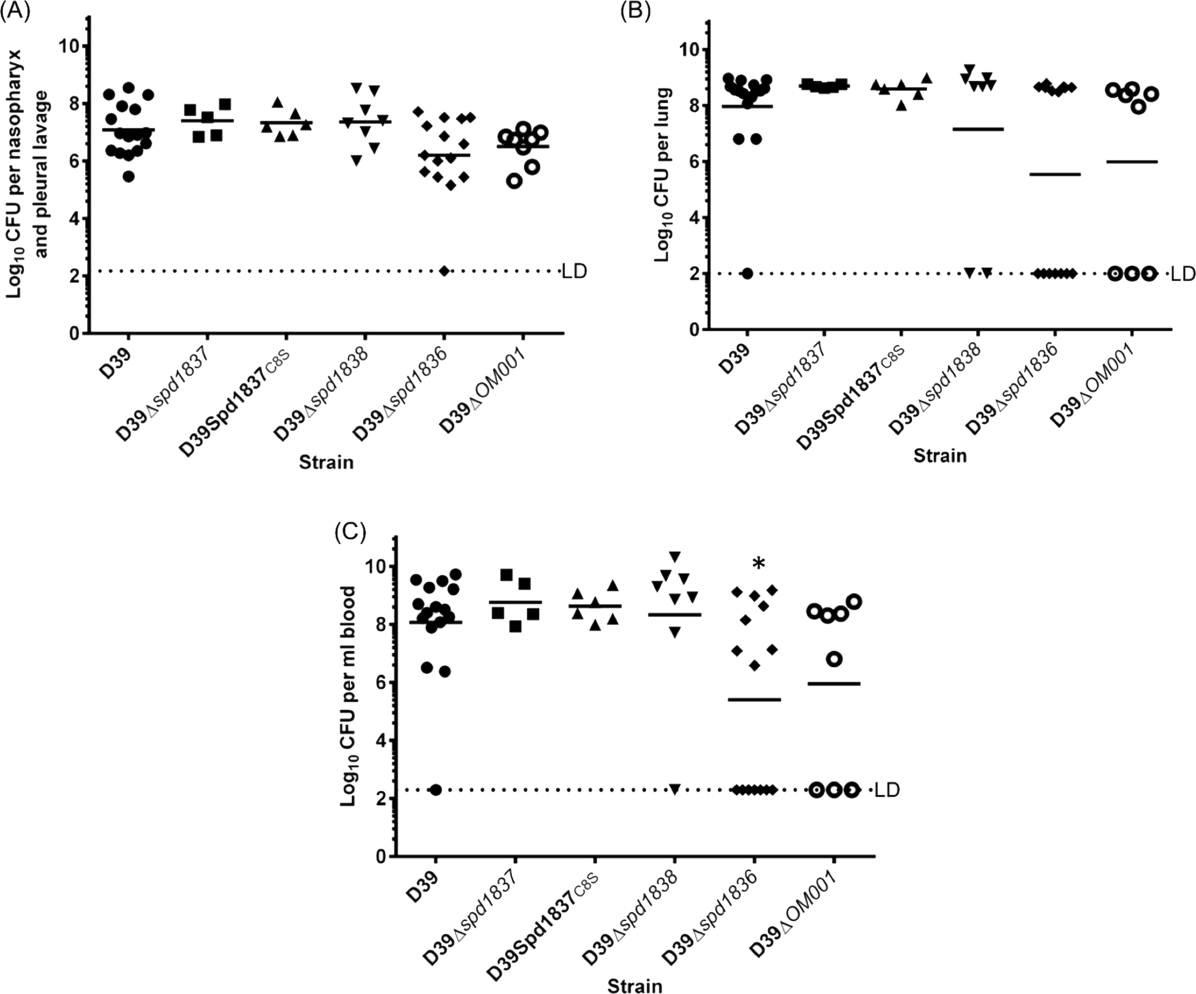
Infection of mice with D39 bacteria and their mutant derivatives. Mice were challenged with D39, D39Δ*spd1837*, D39Spd1837_C8S,_ D39Δ*spd1838*, D39Δ*spd1836* and D39Δ*OM001*. Bacteria were enumerated from the nasopharynx and pleural lavage (A), lungs (B) and blood (C) of each mouse at 48 h post-infection (n = at least 5 per group). Horizontal line represents geometric mean; horizontal broken line denotes limit of detection abbreviated as LD (250 CFU for (A), 100 CFU for (B) and 50 CFU for (C)). *, P < 0.05; Statistical significance was calculated on log-transformed data using Mann Whitney tests. The incidence of pneumococcal invasion into the lungs and blood of mice were compared using two-tailed Fisher’s exact test.

Previous work has shown that the *OM001* operon encoding *spd1836*, *spd1837* and *spd1838* plays a role in pneumococcal virulence [12]. We then undertook animal experiments to investigate the contribution of the individual genes of the operon to virulence in mice using an intranasal model. We found that none of the groups challenged with D39Δ*spd1837*, D39Spd1837_C8S,_ D39Δ*spd1838*, D39Δ*spd1836* and D39*ΔOM001* showed statistically reduced number of bacteria recovered from the nasopharynx, pleural lavage and lungs compared to the group challenged with the wildtype D39 (Fig 3A and 3B). There was however a significant reduction in the number of pneumococci recovered in the blood of mice challenged with D39Δ*spd1836* compared to the wildtype D39 (Fig 3C). Although not reaching statistical significance, a similar trend towards reduced number of bacteria recovered from the nasopharynx, pleural lavage, and lungs was observed for the group challenged with D39Δ*spd1836* and D39*ΔOM001*, and D39Δ*OM001* from the blood compared to the group challenged with the wildtype D39 (Fig 3). Therefore, invasion of the lungs and blood was also compared by Fisher’s exact test. Using this test, we found that significantly fewer mice succumbed with invasive disease of lungs and blood when challenged with D39Δ*spd1836* and D39*ΔOM001* compared to the wildtype D39. Eight out of fifteen mice challenged with D39Δ*spd1836* and three out of eight mice challenged with D39Δ*OM001* had negligible number of pneumococci recovered from their lungs and blood while only one out of sixteen mice challenged with the wildtype D39 did not succumb to invasive disease. Thus, this showed that the contribution of *OM001* operon to pneumococcal virulence was solely due to *spd1836*.

### Spd1836 and Spd1838 may be essential for pneumococcal survival in human saliva

**Fig 4:**
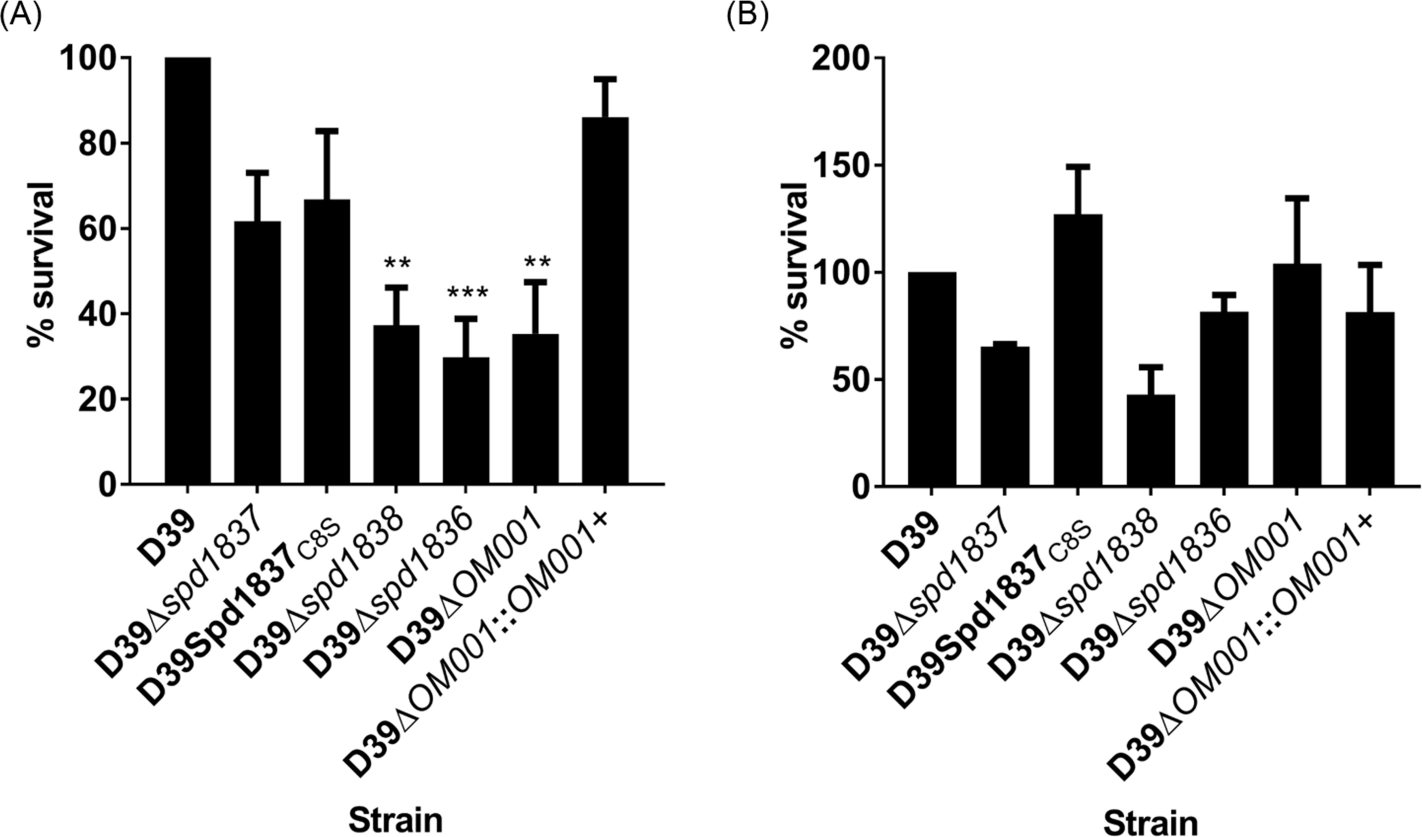
Survival of D39 bacteria and mutant derivatives in human saliva. A starting concentration of 10^6^ CFU mL^-1^ wildtype or mutant bacteria were incubated with saliva at the two conditions; (A) at 25°C without CO_2_ and (B) at 37°C with CO_2_. Experiments were performed in duplicate and repeated three times, independently. There was an approximately 0.5-log decrease in viable count for the D39 strain grown at 25°C without CO_2_ and approximately 2-log decrease in viable count for the D39 strain grown at 37°C with CO_2_. The CFU for D39 at t = 24 at 25°C without CO_2_ was 5 × 10^5^ and the CFU for D39 at t = 24 at 37°C with CO_2_ was 1 × 10^4^. Data were normalized such that the values represent the survival percentage of the mutant strains relative to the wildtype D39 (taken as 100%) ± SEM. Statistical differences between survival of *S*. *pneumoniae* in multiple dilutions of saliva were assessed by one-way ANOVA and Dunnett’s post hoc tests. **, P < 0.01, ***, P < 0.001.

As previous work by Verhagen *et*. *al*. [13] had suggested that the *OM001* operon may play a role in the survival in human saliva, we investigated if our defined *spd1836*, *spd1837* and *spd1838* mutants showed less survival in saliva compared to the wildtype D39 strain. Deletion of *spd1836*, *spd1838* and the overall deletion of the operon *OM001* resulted in lower bacterial survival when grown in human saliva at 25°C without CO_2_ compared to the wildtype D39, and complementation of *OM001* into D39Δ*OM001* restored the survival percentage to wildtype level (Fig 4A). In contrast, none of the mutants including the complemented strain showed significant differences in survival when incubated in human saliva at 37°C with CO_2_ compared to the wildtype strain (Fig 4B). Notably, neither chromosomal deletion nor the active site point mutation of *spd1837* (*spd1837*_*C8S*_) affected pneumococcal survival at 25°C without CO_2_ and at 37°C with CO_2_ (Fig 4A and 4B). Overall, the results suggest that deletion in *spd1836* and *spd1838* reduced pneumococcal survival in human saliva during conditions that mimic transmission (at 25°C without CO_2_), but not during conditions that mimic in-host carriage (37°C with CO_2_). With evidence that human saliva can be a potential reservoir for the person to person spread of the pneumococcus, this would identify these factors as novel factors potentially important for pneumococcal transmission.

## Discussion

While the *OM001* is highly conserved amongst pneumococcal strains, little is known concerning the roles that the individual genes play in the physiology and virulence of the pneumococcus. Previously we have shown that Spd1837 is a PTP of the LMWPTP family (Ahmad *et*. *al*., submitted for publication), and as data suggested importance of the operon in virulence [12], we investigated the individual characteristics of the mutants of these three genes in the operon.

Protein tyrosine phosphorylation in bacteria is now recognized as a critical post-translational regulation of virulence, modulating the pathogenic ability of a range of important human pathogens [9, 37]. The pneumococcus is one of the pathogens for which tyrosine phosphorylation plays an important role, regulating the biosynthesis of its single most important virulence factor, the CPS. The PTP CpsB, has been shown to be required for complete pneumococcal encapsulation [7]. Therefore, we investigated if the LMWPTP Spd1837 and its adjacent co-transcribed proteins, Spd1836 and Spd1838 play a role in the biosynthesis of CPS in the pneumococcus. However, neither Spd1837 nor Spd1836 and Spd1838 modulated tyrosine phosphorylation or CPS production under either aerobic or anaerobic conditions unlike CpsB [38], although the cumulative effects of deleting *spd1836*, *spd1837* and *spd1838* resulted in a slight increase in the levels of cell wall-associated CPS compared to the wildtype. This is perhaps unsurprising for the LMWPTP Spd1837 as PTPs not co-transcribed with bacterial tyrosine kinases generally have species specific roles (S3 and S4 Tables).

As a deletion mutation in the *OM001* operon was previously reported to attenuate pneumococcal virulence in multiple *in vivo* models of infection [12], we investigated the individual contributions of *spd1836*, *spd1837*, and *spd1838* to pneumococcal virulence. Similar to the previous study, we found that the deletion of the operon *OM001* led to a reduction in *in vivo* virulence with fewer mice succumbing to invasive disease of the lungs and blood. However, our data suggested that it was *spd1836* absence rather than the combination of *spd1836*, *spd1837* and *spd1838* deletion that led to the reduced invasive capacity of the pneumococcus, with the D39Δ*spd1836* mutant showing similar results as the D39Δ*OM001* mutant. Our decrease in virulence were not as dramatic an attenuation as seen with the deletion of the operon *OM001* previously [12], however, the prior study utilized different models including gerbils. Regardless, our study identified Spd1836 as a novel virulence factor, playing a role in invasive disease of lungs and blood.

Based on Tn-seq conducted by Verhagen and colleagues [13], the *spd1836* and *spd1837* genes (locus tag SP195_1980 and SP195_1981 respectively in the previous study) were implicated as being potentially important for pneumococcal transmission, however, no testing of individual mutants was reported. Here, we have shown that Spd1836, and Spd1838 along with the operon as a whole play a role in the survival of pneumococci in human saliva, with respective mutants showing statistically significant decreases in CFU when incubated at 25°C without CO_2_ but not when incubated at 37°C with CO_2._ These results are slightly different to those found by the previous study, as we did not see any difference in D39Δ*spd1837* and our differences were only seen in conditions which mimic transmission (25°C without CO_2_). However, our study using defined mutants (rather than the Tn-seq) in a different strain (serotype 2 D39 vs serotype 19F) allowed for a more detailed analysis of the characteristics of these mutants. It would be interesting to investigate the precise role of Spd1838 and Spd1836 proteins in transmission via saliva, given their effects on pneumococcal survival in saliva as reported here.

The epidemiological evidence following vaccine administration highlights the importance of studying transmission and colonization which was previously overlooked in favour of virulence and invasion studies. Given the recent advances in pneumococcal transmission studies [39, 40], one can expect more factors important for transmission will be characterized in the future. We are currently working to identify the mechanisms by which genes of this operon modulate virulence and transmission of the pneumococcus. Additionally, Spd1836 emerges from our study to be a previously uncharacterized virulence factor that may be important for progression to invasive pneumococcal disease. Further work is needed to identify the mechanism for this, and to identify whether this presents as a novel target for the development of new antimicrobials.

## Supporting information

S1 Fig Schematic representation of *OM001* operon. In the chromosome, the operon consists of *spd1838* which encodes for a translocase, YajC (99 amino acids); *spd1837* which encodes for a low molecular weight protein tyrosine phosphatase (142 amino acids); and *spd1836* which encodes for a Membrane Occupation and Recognition Nexus (MORN) repeats-containing protein (136 amino acids).
(TIF)

S2 Fig Growth profiles of D39 strains. Growth curves of *S*. *pneumoniae* strains grown in THY. Data are mean ± SEM absorbance measurements from three independent biological experiments.
(TIF)

S3 Fig Viable bacterial count of the strains at different time points after incubation in human saliva. A starting concentration of 10^6^ CFU mL^-1^ of wildtype or mutant strain was incubated with saliva at two conditions, at 25°C without CO_2_ (A) and at 37°C with CO_2_ (B). Samples were taken for CFU count at t = 0 and 3 hr. Data are mean ± SEM from three independent biological experiments.
(TIF)

S1 Table List of oligonucleotides used. Sequence of oligonucleotides were derived from the chromosomal DNA sequence of *S*. *pneumoniae* serotype 2 D39 and serotype 3 WU2.
(PDF)

S2 Table The total number of mice and the number of surviving mice at the end of an intranasal challenge experiment with the strains. (PDF)

S3 Table LMWPTP-bacterial tyrosine kinase (BY-kinase) pair with a role in capsular polysaccharide (CPS)/exopolysaccharide (EPS) biosynthesis. (PDF)

S4 Table LMWPTP with role(s) in processes other than capsular polysaccharide (CPS)/exopolysaccharide (EPS) biosynthesis. (PDF)
