# Supplementary material for: Role of Streptococcus pneumoniae OM001 operon in capsular polysaccharide production, virulence and survival in human saliva
Source: PLoS One. 2018 Jan 2;13(1):e0190402. doi: 10.1371/journal.pone.0190402 (PMC5749783; doi:10.1371/journal.pone.0190402)
Supplement: S1 Table — Sequence of oligonucleotides were derived from the chromosomal DNA sequence of S. pneumoniae serotype 2 D39 and serotype 3 WU2. (PDF) [file pone.0190402.s004.pdf]

Oligonucleotide	Sequence	Purpose
ZA3	5′-ATACTTACGTTATCTGTGG-3′	F, amplify *spd1837* upstream region
ZA4	5′-AAGAAGGCATTGTAAACGTCCCCG-3′	R, amplify *spd1837* downstream region
ZA5	5′-GGAAAGGGGCCCAGGTCTCTGAAAAGGAGAGTTAAGGTGGAAAATC-3′	F, for overlap extension PCR of *spd1837*, complimentary to *janus cassette*
ZA6	5′-CATTATCCATTAAAAATCAAACGGCCCATTTCCTTTCTTTTATAGAAAAACGG-3′	R, for overlap extension PCR of *spd1837*, complimentary to *janus cassette*
ZA9	5′-GTCTTTGTCAGTCTGGGAAATATTTG-3′	F, exchange *spd1837* C8S, complimentary to upstream of *spd1837*
ZA10	5′-CAAATATTTCCCAGACTGACAAAGAC-3′	R, exchange *spd1837* C8S, complimentary to downstream of *spd1837*
ZA15	5′- GGAAAGGGGCCCAGGTCTCTAGAAGGCGCAATTGAAAAATAAGACG-3′	F, for overlap extension PCR of *spd1838*, complimentary to *janus cassette*
ZA16	5′- CATTATCCATTAAAAATCAAACGGTGTTTTCTCCTTTGTCTTTTACATAGG-3′	R, for overlap extension PCR of *spd1838*, complimentary to *janus cassette*
ZA17	5′- CAAAGGAGAAAACAAGAAGGCGCAATTGAAAAATAAGACG-3′	F, delete *spd1838*, complimentary to upstream of *spd1838*
ZA18	5′- CAATTGCGCCTTCTTGTTTTCTCCTTTGTCTTTTACATAGG-3′	R, delete *spd1838*, complimentary to downstream of *spd1838*
ZA19	5′-GGAAAGGGGCCCAGGTCTCTAGCCTCCTTATCAAAGGAGGTATTAT-3′	F, for overlap extension PCR of *spd1836*, complimentary to *janus cassette*
ZA20	5′-CATTATCCATTAAAAATCAAACGGCTTAACTCTCCTTTTCTAAACGTTC-3′	R, for overlap extension PCR of *spd1836*, complimentary to *janus cassette*
ZA21	5′-GAAAAGGAGAGTTAAGAGCCTCCTTATCAAAGGAGGTATTAT-3′	F, delete *spd1836*, complimentary to upstream of *spd1836*
ZA22	5′-GATAAGGAGGCTCTTAACTCTCCTTTTCTAAACGTTC-3′	R, delete *spd1836*, complimentary to downstream of *spd1836*

ZA24	5′- CAAAGGAGAAAACAAGCCTCCTTATCAAAGGAGGTATTAT-3′	F, delete *OM001*, complimentary to upstream of *spd1836*
ZA25	5′- GATAAGGAGGCTTGTTTTCTCCTTTGTCTTTTACATAGG-3′	R, delete *OM001*, complimentary to downstream of *spd1838*
AS113	5′- CCGTTTGATTTTTAATGGATAATG-3′	F, amplify *janus cassette*
AS114	5′- AGAGACCTGGGCCCCTTTCC-3′	R, amplify *janus cassette*
AS120	5′-TGTTCCCAGCTATTTTTATTCAGA-3′	F, amplify *rpsl*
AS121	5′-TCTCTTTATCCCCTTTCCTTATGC-3′	R, amplify *rpsl*
ZA36	5′- CAGCTAAATTACCAACCTTCC-3′	F, 1 kb upstream of *spd1838*, to amplify *OM001* for complementation
ZA37	5′- TTTTCAACATAAGCTGGAACGTTTC-3′	R, 1 kb downstream of *spd1836*, to amplify *OM001* for complementation

Forward and reverse primers are represented by plus (F) or minus (R), respectively
