# Supplementary material for: Role of Streptococcus pneumoniae OM001 operon in capsular polysaccharide production, virulence and survival in human saliva
Source: PLoS One. 2018 Jan 2;13(1):e0190402. doi: 10.1371/journal.pone.0190402 (PMC5749783; doi:10.1371/journal.pone.0190402)
Supplement: S2 Table — (PDF) [file pone.0190402.s005.pdf]

Group	Total number of mice	Number of surviving mice
D39	16	1
D39Δ*spd1837*	5	0
D39Spd1837$_{C8S}$	6	0
D39Δ*spd1838*	8	1
D39Δ*spd1836*	15	7
D39Δ*OM001*	8	3
