# Supplementary material for: Role of Streptococcus pneumoniae OM001 operon in capsular polysaccharide production, virulence and survival in human saliva
Source: PLoS One. 2018 Jan 2;13(1):e0190402. doi: 10.1371/journal.pone.0190402 (PMC5749783; doi:10.1371/journal.pone.0190402)
Supplement: S3 Table — (PDF) [file pone.0190402.s006.pdf]

S3 Table. LMWPTP-bacterial tyrosine kinase (BY-kinase) pair with a role in capsular polysaccharide (CPS)/exopolysaccharide (EPS) biosynthesis.

Bacteria	LMWPTP	BY-kinase	Function	References
E.coli K-30	Wzb	Wzc	Group 1 CPS assembly	[1]
E. coli K-12	Wzb	Wzc	Colanic acid production	[2]
Enteropathogenic *E. coli*	Etp	Etk	Secretion and assembly of the group 4 CPS	[3, 4]
Acinetobacter iwoffii	Wzb	Wzc	Emulsan production	[5]
Acinetobacter johnsonii	Ptp	Ptk	Colanic acid/EPS synthesis	[6]
Erwinia amylovora	AsmI	AsmH	Amylovoran production	[7]
Klebsiella pneumoniae	Yor5/ Wzb	Yco6/Wzc	CPS production	[8]
Pseudomonas solanacearum	EpsP	EpsK	EPS I production	[9]

References:

1. Wugeditsch T, Paiment A, Hocking J, Drummelsmith J, Forrester C, Whitfield C. Phosphorylation of Wzc, a tyrosine autokinase, is essential for assembly of group 1 capsular polysaccharides in *Escherichia coli*. J Biol Chem. 2001;276(4):2361-71. Epub 2000/10/29. doi: 10.1074/jbc.M009092200. PubMed PMID: 11053445.

2. Vincent C, Duclos B, Grangeasse C, Vaganay E, Riberty M, Cozzone AJ, et al. Relationship between exopolysaccharide production and protein-tyrosine phosphorylation in Gram-negative bacteria. J Mol Biol. 2000;304(3):311-21. Epub 2000/11/25. doi: 10.1006/jmbi.2000.4217. PubMed PMID: 11090276.

3. Ilan O, Bloch Y, Frankel G, Ullrich H, Geider K, Rosenshine I. Protein tyrosine kinases in bacterial pathogens are associated with virulence and production of exopolysaccharide. Embo J. 1999;18(12):3241-8. Epub 1999/06/16. doi: 10.1093/emboj/18.12.3241. PubMed PMID: 10369665; PubMed Central PMCID: PMCPmc1171405.

4. Peleg A, Shifrin Y, Ilan O, Nadler-Yona C, Nov S, Koby S, et al. Identification of an *Escherichia coli* operon required for formation of the O-antigen capsule. J Bacteriol. 2005;187(15):5259-66. Epub 2005/07/21. doi: 10.1128/jb.187.15.5259-5266.2005. PubMed PMID: 16030220; PubMed Central PMCID: PMCPmc1196049.

5. Nakar D, Gutnick DL. Involvement of a protein tyrosine kinase in production of the polymeric bioemulsifier emulsan from the oil-degrading strain *Acinetobacter iwoffii* RAG-1. J Bacteriol. 2003;185(3):1001-9. Epub 2003/01/21. PubMed PMID: 12533476; PubMed Central PMCID: PMCPmc142800.

6. Grangeasse C, Doublet P, Vincent C, Vaganay E, Riberty M, Duclos B, et al. Functional characterization of the low-molecular-mass phosphotyrosine-protein phosphatase of

Acinetobacter johnsonii. J Mol Biol. 1998;278(2):339-47. Epub 1998/06/10. doi: 10.1006/jmbi.1998.1650. PubMed PMID: 9571056.

7. Bugert P, Geider K. Characterization of the *amsI* gene product as a low molecular weight acid phosphatase controlling exopolysaccharide synthesis of *Erwinia amylovora*. FEBS Lett. 1997;400(2):252-6. Epub 1997/01/03. PubMed PMID: 9001408.

8. Preneta R, Jarraud S, Vincent C, Doublet P, Duclos B, Etienne J, et al. Isolation and characterization of a protein-tyrosine kinase and a phosphotyrosine-protein phosphatase from *Klebsiella pneumoniae*. Comp Biochem Physiol B Biochem Mol Biol. 2002;131(1):103-12. Epub 2001/12/18. PubMed PMID: 11742763.

9. Huang J, Schell M. Molecular characterization of the eps gene cluster of *Pseudomonas solanacearum* and its transcriptional regulation at a single promoter. Mol Microbiol. 1995;16(5):977-89. Epub 1995/06/01. PubMed PMID: 7476194.
