# Supplementary material for: Role of Streptococcus pneumoniae OM001 operon in capsular polysaccharide production, virulence and survival in human saliva
Source: PLoS One. 2018 Jan 2;13(1):e0190402. doi: 10.1371/journal.pone.0190402 (PMC5749783; doi:10.1371/journal.pone.0190402)
Supplement: S4 Table — (PDF) [file pone.0190402.s007.pdf]

S4 Table. LMWPTP with role(s) in processes other than capsular polysaccharide (CPS)/exopolysaccharide (EPS) biosynthesis.

Bacteria	LMWPTP	Function	References
Porphyromonas gingivalis	Ltp1	Regulate transcriptional activity of the global regulator LuxS	[1]
Bacillus subtilis	YfkJ	Regulate ethanol resistance	[2]
Burkholderia contaminans	BceD	Biofilm formation	[3, 4]
E. coli	Etp	Regulate heat shock resistance	[5]
Mycobacterium tuberculosis	PtpA	Inhibit phagosome acidification and block fusion with lysosomes	[6-8]
Burkholderia cenocepacia	Dpm	Inhibit phagosome maturation	[9]

References:

1. Maeda K, Tribble GD, Tucker CM, Anaya C, Shizukuishi S, Lewis JP, et al. A *Porphyromonas gingivalis* tyrosine phosphatase is a multifunctional regulator of virulence attributes. Mol Microbiol. 2008;69(5):1153-64. Epub 2008/06/25. doi: 10.1111/j.1365-2958.2008.06338.x. PubMed PMID: 18573179; PubMed Central PMCID: PMCPmc2537464.

2. Musumeci L, Bongiorni C, Tautz L, Edwards RA, Osterman A, Perego M, et al. Low-molecular-weight protein tyrosine phosphatases of *Bacillus subtilis*. J Bacteriol. 2005;187(14):4945-56. Epub 2005/07/05. doi: 10.1128/jb.187.14.4945-4956.2005. PubMed PMID: 15995210; PubMed Central PMCID: PMCPmc1169535.

3. Ferreira AS, Leitao JH, Sousa SA, Cosme AM, Sa-Correia I, Moreira LM. Functional analysis of *Burkholderia cepacia* genes *bceD* and *bceF*, encoding a phosphotyrosine phosphatase and a tyrosine autokinase, respectively: role in exopolysaccharide biosynthesis and biofilm formation. Appl Environ Microbiol. 2007;73(2):524-34. Epub 2006/11/23. doi: 10.1128/aem.01450-06. PubMed PMID: 17114319; PubMed Central PMCID: PMCPmc1796985.

4. Ferreira AS, Silva IN, Fernandes F, Pilkington R, Callaghan M, McClean S, et al. The tyrosine kinase BceF and the phosphotyrosine phosphatase BceD of *Burkholderia contaminans* are required for efficient invasion and epithelial disruption of a cystic fibrosis lung epithelial cell line. Infect Immun. 2015;83(2):812-21. doi: 10.1128/iai.02713-14. PubMed PMID: 25486990.

5. Klein G, Dartigalongue C, Raina S. Phosphorylation-mediated regulation of heat shock response in *Escherichia coli*. Mol Microbiol. 2003;48(1):269-85. Epub 2003/03/27. PubMed PMID: 12657060.

6. Bach H, Papavinasasundaram KG, Wong D, Hmama Z, Av-Gay Y. *Mycobacterium tuberculosis* virulence is mediated by PtpA dephosphorylation of Human Vacuolar Protein Sorting 33B. Cell Host & Microbe. 2008;3(5):316-22. Epub 2008/05/14. doi: 10.1016/j.chom.2008.03.008. PubMed PMID: 18474358.

7. Poirier V, Bach H, Av-Gay Y. *Mycobacterium tuberculosis* promotes anti-apoptotic activity of the macrophage by PtpA protein-dependent dephosphorylation of host GSK3α. J

Biol Chem. 2014;289(42):29376-85. Epub 2014/09/05. doi: 10.1074/jbc.M114.582502. PubMed PMID: 25187516; PubMed Central PMCID: PMCPmc4200286.

8. Wong D, Bach H, Sun J, Hmama Z, Av-Gay Y. *Mycobacterium tuberculosis* protein tyrosine phosphatase (PtpA) excludes host vacuolar-H^+-ATPase to inhibit phagosome acidification. Proc Natl Acad Sci U S A. 2011;108(48):19371-6. Epub 2011/11/17. doi: 10.1073/pnas.1109201108. PubMed PMID: 22087003; PubMed Central PMCID: PMCPmc3228452.

9. Andrade A, Valvano MA. A *Burkholderia cenocepacia* gene encoding a non-functional tyrosine phosphatase is required for the delayed maturation of the bacteria-containing vacuoles in macrophages. Microbiology. 2014;160(Pt 7):1332-45. Epub 2014/04/15. doi: 10.1099/mic.0.077206-0. PubMed PMID: 24728272.
